# Asymptomatic 3-methylglutaconic aciduria type 1 detected by high C5-OH on newborn screening

**DOI:** 10.1016/j.ymgmr.2023.101024

**Published:** 2023-11-22

**Authors:** Tomoyo Itonaga, Miwako Maeda, Hiroshi Koga, Yuki Hasegawa, Kenji Ihara

**Affiliations:** a Department of Pediatrics, Oita University Faculty of Medicine, 1-1 Idaigaoka, Hasama, Yufu, Oita 879-5593, Japan; b Department of Pediatrics, National Hospital Organization Beppu Medical Center, 1473 Oaza-Uchikamado, Beppu, Oita 874-0011, Japan; c Department of Pediatrics, Japanese Red Cross Matsue Hospital, 200 Horomachi, Matsue, Shimane 690-8506, Japan

**Keywords:** 3-methylglutaconic aciduria type 1 (MGGA1), C5-OH, Newborn screening, Asymptomatic, *AUH* gene

## Abstract

3-Methylglutaconic aciduria type 1 (MGCA1) is an inborn error of leucine catabolism caused by pathogenic variants of the *AUH* gene. MGCA1 can be identified by newborn screening (NBS) with elevated C5-OH levels. We herein report a girl with MGCA1 detected by NBS. On day 5 after birth, NBS detected high C5-OH levels of 1.17 μmol/L (1.56 μmol/L [retest]). A urinary organic acid analysis revealed the abnormal excretion of 3-methylglutaconic, 3-methylglutaric, and 3-hydroxyisovaleric acids. Two novel heterozygous loss-of-function variants in the *AUH* gene were identified by genetic testing. We observed the patient without any treatment, such as a leucine-restricted diet. She had episodes of febrile illness several times in infancy but did not develop febrile convulsions or encephalopathy. She is now two years and six months old, and her physical growth and psychomotor development have progressed normally. In a review of the literature and our case, four children with MGCA1 identified during the neonatal period were asymptomatic or exhibited speech delay, regardless of whether or not they had been managed with specific treatments. Treatments such as dietary leucine restriction and carnitine supplementation may have little effect on MGCA1 in childhood; however, further investigation is warranted to evaluate the benefits of specific treatments to prevent potential long-term neurological complications.

## Highlights

• 3-Methylglutaconic aciduria type 1 (MGCA1) is caused by pathogenic variants of the *AUH* gene, leading to leucine metabolism disorders.
• Newborn screening identified MGCA1 through elevated C5-OH levels and novel variants in the *AUH* gene detected in a case study.
• MGCA1 cases show heterogeneous symptoms and severity; current treatments such as leucine restriction might not have a significant impact on growth and development during childhood.

## 1 Introduction

Leucine catabolism mainly occurs in the mitochondria, and genetic abnormalities in the first step of this process are recognized to cause maple syrup urine disease and isovaleric acidemia. These diseases are relatively common inborn errors of leucine catabolism, and their management and treatment are well-established.

The enzymatic activity of 3-methylglutaconyl CoA hydratase (MGH) catalyzes the fifth step in the leucine degradation pathway and the conversion of 3-methylglutaconyl CoA to HMG-CoA. The genetic defect in MGH encoded by the AU-specific RNA-binding protein (*AUH*) gene has been assigned to 3-methylglutaconic aciduria type 1 (MGCA1) (MIM ID #250950), characterized by marked urinary excretion of 3-methylglutaconic acid (3-MGA) along with urinary 3-methylglutaric acid (3-MG) and 3-hydroxyisovaleric acid (3-HIVA) excretion [[1], [2], [3]].

The clinical spectrum of MGCA1 is highly variable, ranging from asymptomatic to neurologically symptomatic childhood. Mild neurological impairments, such as speech delay, quadriplegia, and dystonia, have been reported as pediatric symptoms, whereas slowly progressive leukoencephalopathy or severe encephalopathy with basal ganglia involvement are distinctive manifestations in adulthood [1,[4], [5], [6], [7], [8], [9], [10], [11]]. In addition, non-neurological symptoms, such as dilated cardiomyopathy [12] and central precocious puberty [13] have also been reported. The organ-specific abnormalities associated with MGCA1 are speculated to result from disrupted mitochondrial function and the accumulation of toxic metabolites in the affected tissues.

Elevated levels of 3-hydroxyisovalerylcarnitine (C5-OH), an intermediate metabolite of 3-methycrotonyl-CoA, are typically observed in the blood or urine of patients with MGCA1. Therefore, MGCA1 can be identified by newborn screening (NBS), and several cases of asymptomatic MGCA1 have been reported. However, an evidence-based consensus treatment has not yet been established.

We herein report a neonate with MGCA1 identified based on the elevation of C5-OH on NBS. She has been asymptomatic with normal physical growth and psychomotor development for two years and six months without any treatment, such as a leucine-restricted diet or carnitine supplementation.

## 2 Case presentation

**Fig. 1:**
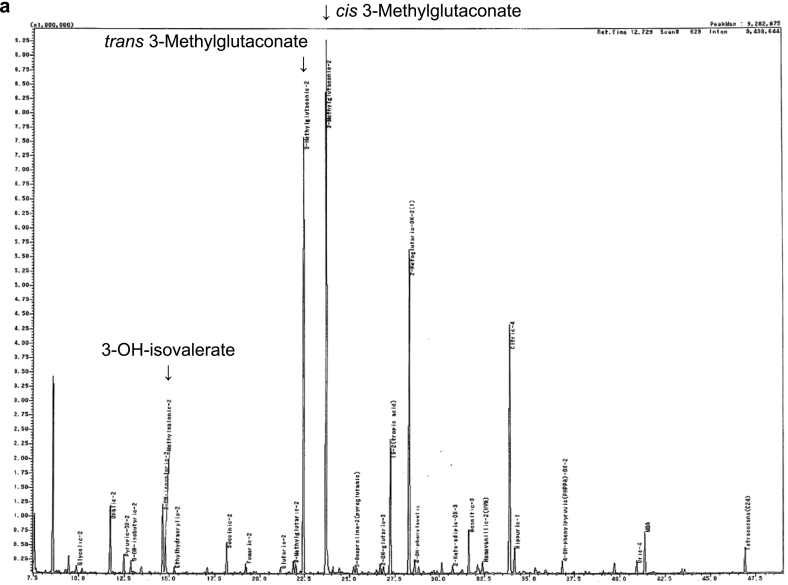

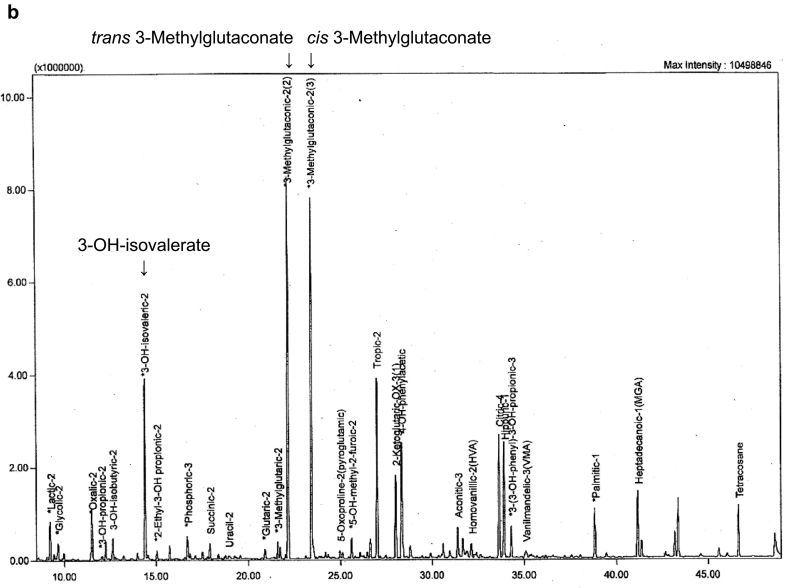
Electropherograms of urinary gas chromatography–mass spectrometry. a. Urinary 3-MGA, 3-MG and 3-HIVA were remarkably high at neonatal period. b. Urinary 3-MGA, 3-MG and 3-HIVA were still high at the age of 2 years.

The patient was born at 37 weeks of gestation as the second child of healthy, nonconsanguineous parents. Her birth weight was 2676 g (+0.15 standard deviations [SD]), and her length was 46.5 cm (−0.35 SD). She had no family history of sudden death or developmental delays. NBS performed on day 5 showed an elevation of C5-OH levels at 1.17 μmol/L, and re-testing also showed a high level of C5-OH (1.56 μmol/L). Laboratory data did not show elevated blood ammonia, glucose, free fatty acids, or ketone bodies levels. Gas chromatography-mass spectrometry revealed a marked increase in 3-MGA and moderate increases in 3-MG and 3-HIVA in the urine (Fig. 1a). Echocardiography did not reveal cardiomyopathy. Two novel heterozygous loss-of-function variants in *AUH* (NM_001698.3: c.636_645del [frameshift variant] and c.262+1G>T [splice variant]) were detected by genetic testing. These variants have not been registered in either ClinVar (https://www.ncbi.nlm.nih.gov/clinvar/) or the Human Genome Mutation Database (www.hgmd.org), and they are very rare because they are not included in the public databases of minor allele frequencies, such as gnomAD (v4.0.0, https://gnomad.broadinstitute.org/) and 8.3KJPN, which include 8300 Japanese reference genomes (https://jmorp.megabank.tohoku.ac.jp/202102/). The frameshift variant (c.636_645del) results in an interrupted translation. *In silico* prediction scores for the splice valiant (c.262+1G>T) also supported the interruption of the splice site (splice AI 0.99: donor cite loss, Pangolin scores 0.81: splice loss). Based on this observation, we considered both of the variants to be pathogenic. Consequently, the patient was diagnosed with MGCA1 due to compound heterozygous pathogenic variants of the *AUH* gene. The genetic analysis of the parents has not been carried out because of a lack of informed consent from the parents.

We subsequently managed her without any specific treatment, such as a leucine-restricted diet and carnitine administration. She had episodes of febrile illness, including exanthem subitum, several times in infancy but did not develop febrile convulsions or encephalopathy. Brain MRI and echocardiography at one year and seven months old showed no abnormal findings. She passed all items of the modified checklist for autism in toddlers (M-CHAT).

**Fig. 2:**
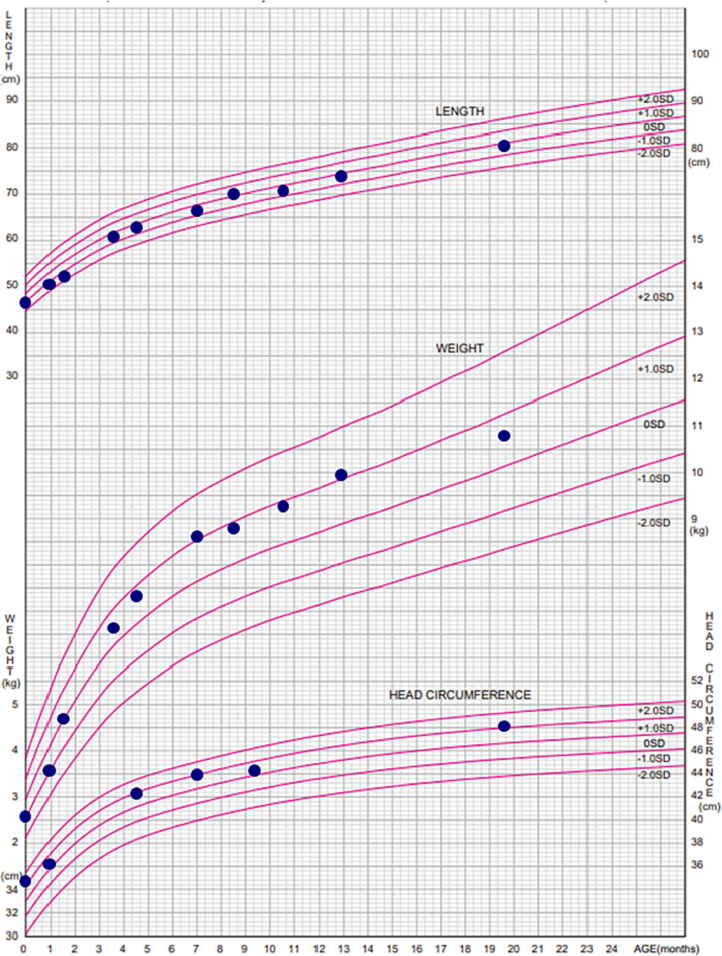
Growth chart. The patient showed normal growth in height, weight, and head circumference.

She is currently two years and six months old under normal growth conditions (Fig. 2). Her psychomotor development has normally progressed; the developmental quotient at 1 year 7 months old and 2 years 5 months old, as assessed by the *Enjohji Developmental Test in Infancy and Early Childhood*, was 114 and 120, respectively. The levels of urinary 3-MGA, 3-HIVA and blood C5-OH remained remarkably high (Fig. 1b, Table 1).

**Table 1:** Asymptomatic MGCA1 patients at diagnosis reported in the literature.

No.	Gender	Diagnostic clues	C5OH level (μmol/L)		*AUH* variants	Age at last examination	Treatment	Clinical features	References
			at NBS	During follow-up period					
1	M	NBS*	0.3	Normalized (at age of 3 m)	c.80delG/c.80delG	21 y	None	Normal	[11]
2	F	NBS*	ND	ND	c.80delG/c.80delG	19 y	None	Normal	[11]
3	M	NBS	ND	ND	c.620T>G/c.676C>T	18 y	None	Normal	[11]
4	M	NBS	2.5	Normalized (at 2.5 y)	c.80delG/c.80delG	9 y	ND	Normal	[2]
5	M	Sibling**	ND	ND	c.589C>T/c.589C>T	5.4 y	ND	Speech delay	[2,16]
6	F	NBS	0.8, 1.1	3.33 (at age of 2.5 y)	Deletion of exon 2–3 /deletion of exon 8–10	2.5 y	Mild protein restriction, l-carnitine	Speech delay	[10]
7	F	NBS	1.17, 1.56	3.54 (at age of 2.4 y)	c.636_645del/ c.262+1G>T	2.4 y	None	Normal	Our case
8	M	NBS	1.7	ND	c.260del/c.80del	1.3 y	None	Normal	[11]

*Patients 1 and 2 were siblings. **Patient 5 was examined despite being asymptomatic because his elder brother, not listed, showed developmental delay and symptomatic hypoglycemia with metabolic acidosis.

M, male; F, female; NBS, newborn screening; ND, not described; m, month(s); y, year(s).

## 3 Discussion

We herein report a girl with MGCA1 who was diagnosed with elevated C5-OH in NBS. She has been asymptomatic for two years. Notably, her physical growth and psychomotor development progressed normally without any specialized treatment.

The phenotype of MGCA1 in childhood is reported to be highly variable, ranging from asymptomatic to neurologically symptomatic. In addition, recent reports have addressed a late-onset (adult) phenotype of leukoencephalopathy [8,14,15]. Given this knowledge of a late-onset phenotype, the establishment of evidence-based treatment for asymptomatic children merits consideration. Several management strategies, such as dietary restriction, carnitine supplementation, and high-calorie feeding during catabolic stress, have been attempted [6,8]. Although such approaches have the potential to correct biochemical abnormalities and possibly forestall the development of neurological symptoms and related complications, it is difficult to assess the clinical benefits of treatment compared with no treatment.

Seven cases that were asymptomatic at the diagnosis, including our own case, have been reported thus far (Table 1) [2,10,11,16]. Six of the seven cases were detected with high levels of C5-OH on newborn screening. Notably, three patients in the Australian cohort received no treatment and were asymptomatic until adulthood [11]. In the cohort, the authors speculated that MGCA1 might be unrelated to neurological symptoms, as two siblings with both MGCA1 and autism spectrum disorder had another sibling, without MGCA1, who also had autism spectrum disorder [11]. Our literature review was associated with a couple of potential limitations. First, the sample size of the reported cases may have been too small to investigate the rationality of follow-up protocols without specific treatment. Second, this report was unable to demonstrate the long-term outcome of our case, especially concerning neurological assessments and brain MRI findings. Unfortunately, not all case reports provided the requisite information. In addition, nationwide NBS using tandem mass spectrometry just started less than 10 years ago in Japan, and therefore, case reports of asymptomatic MGCA1 patients have been limited.

As mentioned above, an evidence-based consensus treatment has not yet been established. Therefore, several issues also must be considered regarding how management strategies should be introduced for cases of MGCA1. For example, no genotype–phenotype correlations have been reported and the clinical spectrum is heterogeneous. In addition, even if asymptomatic in childhood, patients may later be revealed as having the adult-onset type. In the present case, we planned to share the disease information described above with the parents from time to time, and regular evaluation of physical growth, neuropsychiatric development, and head MRI will be conducted for long-term management.

Currently, NBS has identified individuals with a number of inborn errors of metabolism (IEMs), such as MGCA1, who are asymptomatic without treatment. There is no definitive evidence of effective treatment for some IEMs, which is why MGCA1 has not been screened by NBS in many countries. Nevertheless, the application of tandem mass spectrometry for NBS is expected to increase the number of IEM cases. Worldwide clinical research is needed to investigate the potential clinical application of specific therapies for MGCA1.

## 4 Conclusion

We herein report a girl with MGCA1 who has been asymptomatic for two years without any treatment. Given the absence of an established treatment for MGCA1, there is a need for comprehensive, long-term, global studies to develop evidence-based management approaches for asymptomatic individuals diagnosed by NBS.

## Ethical approval statement

No ethical approval was required as the testing in the patient was performed according to medical indication, as part of standard of care.

## Patient consent

The patient provided written informed consent for this manuscript.

## Financial disclosure

This research did not receive any specific grant from funding agencies in the public, commercial, or not-for-profit sectors.

## Author contributions

All persons who are listed as authors certify that they have participated and take responsibility for the content of the manuscript. Tomoyo Itonaga: conceptualization, writing-original draft preparation. Miwako Meda: writing-review, and editing. Hiroshi Koga: writing - review, and editing. Yuki Hasegawa: writing - review, and editing. Kenji Ihara: supervision, writing - review and editing.

## Declaration of Competing Interest

The authors declare that they have no known competing financial interests or personal relationships that could have influenced the work reported in this study.

## Data Availability

The data that has been used is confidential.
